# Identification of critical links based on the optimal reliable path in stochastic traffic networks

**DOI:** 10.1371/journal.pone.0301272

**Published:** 2024-04-09

**Authors:** Yi Sun, Sirui Wang, Xiang Xu, Liang Shen

**Affiliations:** 1 School of Management, Xuzhou Medical University, Xuzhou, Jiangsu, China; 2 School of Public Health, Xuzhou Medical University, Xuzhou, Jiangsu, China; Al Mansour University College-Baghdad-Iraq, IRAQ

## Abstract

In urban stochastic transportation networks, there are specific links that hold great importance. Disruptions or failures in these critical links can lead to reduced connectivity within the road network. Under this circumstance, this manuscript proposed a novel identification of critical links mathematical optimization model based on the optimal reliable path with consideration of link correlations under demand uncertainty. The method presented in this paper offers a solution to bypass the necessity of conducting a full scan of the entire road network. Due to the non-additive and non-linear properties of the proposed model, a modified heuristic algorithm based on K-shortest algorithm and inequality technical is presented. The numerical experiments are conducted to show that improve a certain road link may not necessarily improve the overall traffic conditions. Moreover, the results indicate that if the travel time reliability is not considered, it will bring errors to the identification of key links.

## 1. Introduction

Urban transportation plays a vital role in fostering urban economic development by facilitating the movement of goods and residents. Nevertheless, this complex system is vulnerable to a range of internal and external factors that can disrupt its smooth operation. Natural disasters, adverse weather conditions, and traffic accidents are among the key risks that can lead to the breakdown of essential connections within the urban road network. The failure of critical links can create a domino effect, overloading other links and setting off a chain reaction of failures across the network. This ripple effect has the potential to escalate into widespread traffic congestion or even the total collapse of the road network, significantly impacting residents’ lives and impeding social productivity.

The rapid urbanization and economic growth have led to a sharp increase in the number of vehicles in cities. This surge often overwhelms the existing transportation infrastructure, causing chronic traffic congestion and frequent accidents like collisions. These incidents can disrupt specific road connections, impeding traffic flow. Severe disruptions can trigger a chain reaction, spreading congestion to nearby areas. Consequently, some parts of the road network face prolonged congestion, reducing its capacity and impacting public travel efficiency and overall quality of life. To tackle these issues, it is crucial for government authorities and transportation departments to identify key links accurately and bolster the road network’s resilience proactively.

Indeed, there have been scholars who have researched methods for identifying key links. Several approaches have been explored to address this issue. Sohn et al. [1] utilized the extent of accessibility loss in the road network to identify critical links during flood disasters in Maryland. Taylor et al. [2] proposed a cost index that incorporates general travel cost, Hansen accessibility index, and the Australian ARIA accessibility index. Jenelius et al. [3] selected indicators based on whether the start and end points of a link are connected. If connected, they evaluated the importance of the link by considering the increase in travel cost between all origin-destination (OD) pairs before and after the link failure. If not connected, the road network was divided into independent parts with infinite cost, and the importance of the link was assessed based on the amount of traffic demand that could not reach its destination. Scott et al. [4] proposed the Network Robustness Index (NRI), which characterizes the impact of network flow, road capacity, and network topology on network efficiency. NRI measures the total change in impedance in the road network before and after a link failure. Sullivan et al. [5] introduced the Network Trip Robustness (NTR) index, an improvement upon NRI. NTR uses cost per trip as the criterion for identifying key links, independent of network size, topology, or connectivity. Balijepalli and Oppong [6] summarized travel cost indicators and network robustness indicators and introduced the Network Vulnerability Index (NVI), which considers the availability of remaining capacity in links. De Oliveira et al. [7] comprehensively selected congestion indicators (e.g., V/C ratio, congestion index), robustness indicators, and improved robustness indicators to create a comprehensive index for identifying key links. Rupi et al. [8] measured the importance of links based on average daily traffic demand and the change in network cost before and after segment deletion. Yang et al. [9] proposed the definition of the critical link for an urban traffic network and establishes mathematical model for determining critical link considering the travelers’ heterogeneous risk-taking behavior. Du et al. [10] presented a new capacity-based network robustness index for identifying critical links and evaluating the transportation system performance. It uses the change of the total network capacity as an evaluation measure. Vodák et al. [11] introduced a rapid deterministic algorithm for identification of the most critical links which are capable of causing network disruptions. Feng et al. [12] proposed a novel identification method of critical roads based on the combination of GPS trajectory data and directed weighted complex network. Li et al. [13] proposed an approach considering the traffic flow betweenness index (TFBI) to identify critical links, which can significantly reduce the computational burden compared with the traditional full-scan method. Almotahari and Yazici [14] introduced Link Criticality Index (LCI) that identifies the criticality ranking within a single User Equilibrium (UE) traffic assignment using Frank-Wolfe (FW) algorithm. Du et al. [15] develop a new link criticality indicator based on the network capacity concept of a multimodal transportation network. Jin et al. [16] developed a rigorous, extensible, mathematical model to identify the critical combination of roads in urban road networks for multiple disruption scenarios. Arabi et al. [17] asked a fundamental question on equity achievement of such measures and develops a new framework to incorporate road users’ vulnerabilities in identifying critical network links.

It is important to note that the indicators mentioned earlier are systematic measures used to identify critical links in a road network. These indicators take into account factors such as network topology and traffic flow, leading to more accurate results. However, conducting a comprehensive scan to calculate these indicators can be extremely time-consuming, especially for extensive road networks (Mattsson and Jenelius [18]). In reality, it may even be impractical due to the computational load it entails.

**Fig 1:**
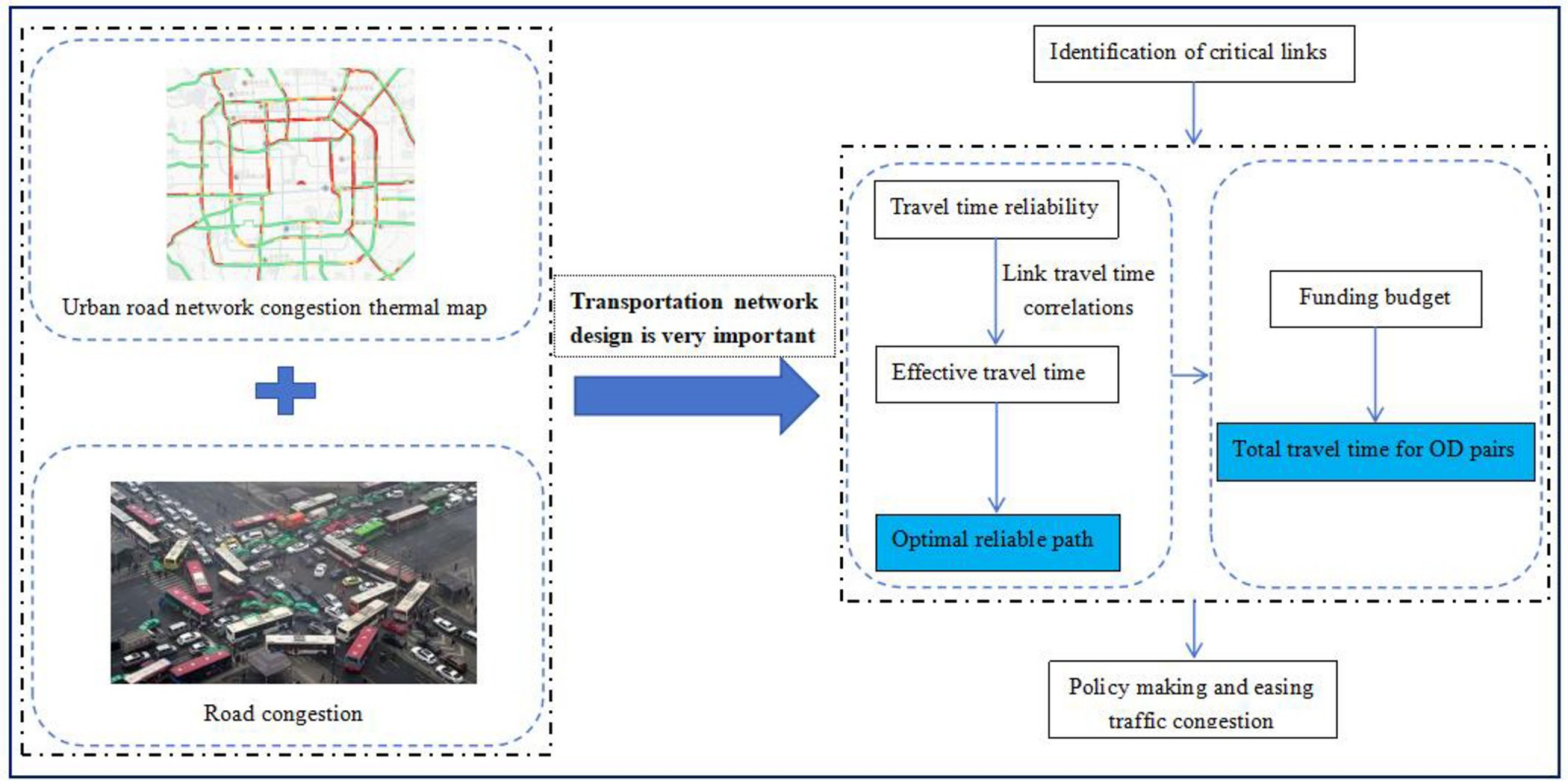
Flow chart of transportation network design.

Under this circumstance, this manuscript proposed a novel identification of critical links mathematical model based on optimal reliable path with consideration of link correlations under uncertainty. The detailed flow chart of transportation network design is shown in Fig 1. The method presented in this paper offers a solution to bypass the necessity of conducting a full scan of the entire road network. As a result, it enhances both computational efficiency and accuracy. In contrast to existing models, this model focuses on selecting the optimal path for each pair under uncertain conditions, taking into account the link correlations. It calculates the estimated time required for all pairs in the entire network. By minimizing the time required for all OD pairs in the network through improvements made to all links, the overall objective of enhancing the entire network is achieved.

The paper is organized as follows: Section 2 introduces the assumptions and presents an optimization model. Section 3 outlines the solution algorithm and presents the flow chart of the proposed model. In Section 4, the experimental results are presented and analyzed. Finally, Section 5 concludes the paper.

## 2. Model assumptions and model establishment

### 2.1. Model assumptions

1. The assumption is made that the OD demand and link flow conform to a normal distribution (Shao et al. [19]).
2. The on-time reliability is equal to or greater than 50% (*θ* ≥ 50%) (Shen et al. [20]).
3. It is assumed that the coefficient of variation for OD demand in the traffic network is equal to the coefficient of variation for path flow.

### 2.2. Model establishment

#### 2.2.1. Definition of travel time reliability

According to Asakura and Kashiwadani [21]’s definition of “travel time reliability”, and in conjunction with the research content of this paper, the following definition is proposed for “reliability”.

**Definition 2.1:** Reliability refers to the probability that a traveler can complete a specified link, path, or OD pair within a given time under certain conditions [22–24].

Based on this definition, travel time reliability can be regarded as the likelihood of completing a path between a specified origin-destination (OD) pair or through a certain link within a designated time frame. The mathematical can be expressed as follows:

$$R\left(t\right)=P\left\{T\leq t\right\}$$ (1)

In the equation, the variable *t* represents the specified time threshold, which is a deterministic value indicating the possible travel time *T* through the link or OD pair. On the other hand, *R*(*t*) is a random variable that represents the reliability of traveling through the link or OD pair within the specified time. In other words, it denotes the probability of successfully completing the path. As per the provided definition, the reliability of travel time refers to the distribution function of the possible travel time through the link or OD pair.

To find an optimal path that meets specific reliability requirements, the concept of effective travel time is introduced. For a particular link *a* between an origin-destination (OD) pair (*r*,*s*), travelers strive to determine a time threshold $t_{a}^{rs}$ that ensures a desired level of “reliability” on that link. In other words, they aim to establish a threshold where the probability *θ* of successfully traversing the link within the specified time is $t_{a}^{rs}$. Based on this definition of “reliability”, the effective travel time can be defined as follows:

**Definition 2.2:** The time-constrained probabilistic model (2) establishes probabilistic constraints on time $t_{a}^{rs}$:

$$\begin{array}{c} t_{a}^{rs}=\min\;{\tilde{t}}_{a}^{rs}\\ \text{s.t.P}\left\{T_{a}^{rs}\leq {\tilde{t}}_{a}^{rs}\right\}\geq \theta . \end{array}$$ (2)

The time $t_{a}^{rs}$ established by the probability constraint model (2) is the effective travel time.

#### 2.2.2. Definition of reliable path

In addition, the total travel time on a path *k* between an OD pair (*r*,*s*) is defined as the sum of the travel times for each link between the OD pair and can be expressed as follows:

$$T_{k}^{rs}=\sum_{a\in E}\delta_{a,k}^{rs}T_{a}^{rs}.$$ (3)

In the equation, $T_{k}^{rs}$ represents the total travel time on the path *k* between an OD pair (*r*,*s*), *E* represents the set of edges in the network graph, and $T_{a}^{rs}$ represents the travel time for link *a*, where

$$\delta_{a,k}^{rs}=\left\{\begin{array}{cc} 1, & if\;a\in k\\ 0, & if\;a\;\notin \;k \end{array}\right.$$ (4)

As stochastic variables cannot be directly compared, the definition of effective travel time serves as a foundation for measuring the superiority of a path by considering its effective travel time. This approach guarantees that the travel time’s “reliability” satisfies specific requirements and enables the search for reliable paths in uncertain conditions. Therefore, we define a “reliable path” as follows:

**Definition 2.3:** A path k^*^ is classified as a reliable path if and only if, when subjected to the same “reliability” requirement, it possesses the shortest effective travel time compared to all other paths between the origin-destination (OD) pair (Clark and Watling [25]). The mathematical model can be depicted as follows:

$$k^{*}=\arg\;\underset{k\in A}{\min}\;t_{k}^{rs}$$ (5)

In the equation, k^*^ represents the set of reliable paths between the OD pair (*r*,*s*), the notation *A* means consists of all the paths connecting the OD pair (*r*,*s*).

#### 2.2.3. The modified mathematical model based on the optimal reliable path with consideration of correlations

The effective travel time for path *k* between OD pair (*r*,*s*) can be expressed as follows:

$$t_{k}^{rs}=\sum_{a\in E}\delta_{a,k}^{rs}\mu_{a}+\Phi^{-1}(\theta )\sqrt{\sum_{a\in E}\delta_{a,k}^{rs}\sigma_{a}^{2}+2\sum_{a<b}\delta_{a,k}^{rs}\delta_{b,k}^{rs}\rho_{ab}\sigma_{a}\sigma_{b}}$$ (6)

Where $t_{k}^{rs}$ denotes the total effective travel time of a path *k* between the OD pair (*r*,*s*). It is a function of various factors including the level of reliability *θ*, the mean travel time *μ*_*a*_ on link *a*, the standard deviation of travel time *σ*_*a*_ on a link *a*, and the correlation coefficient between links *a* and *b*. This implies that the path effective travel time $t_{k}^{rs}$ depends on both the mean and variance of travel times on the links along the path. These parameters collectively determine the reliability and variability of travel time experienced on the path.

In order to find the critical links based on the optimal reliable path, the optimization mathematical is proposed as follows:

$$\begin{array}{l} \sum_{rs}\sum_{a\in E}\min t_{k}^{rs}\left(\Delta \mu_{a},\Delta \sigma_{a}\right) \end{array}$$ (7)

$$s.t.\;k_{a}\left(\Delta \mu_{a}+\Delta \sigma_{a}\right)=M.$$

In this equation, we can denote the mean travel time and variance on a link after improvement as Δ*μ*_*a*_, Δ*σ*_*a*_. Here, *M* represents a fixed value representing the given total budget allocated for improving the performance of the links. These parameters allow us to quantify the expected improvement in travel time and the reduction in variability achieved through the allocated budget. By considering these values, we can evaluate the impact of link improvements on the overall effective travel time and reliability of a path.

Based on the model, it is clear that the total effective travel time $t_{k}^{rs}$ between origin-destination (OD) pair (*r*,*s*) is not merely the sum of individual link travel times. Consequently, traditional optimal path search algorithms are not suitable in this scenario. Additionally, the model takes into account the total travel time for all OD pairs within the entire network and necessitates improvements on various links.

The objective of this study is to observe the outcomes and identify the most critical link. As a result, this research builds upon existing algorithmic approaches and integrates the total travel time for all OD pairs in the network. This process enables the determination of the most significant link in terms of its impact on overall travel time and reliability. By considering a holistic perspective, the study seeks to provide valuable insights for efficient road network improvement and optimization.

## 3. Algorithm design

The proposed model exhibits a specific characteristic, namely that the effective travel time is non-additive due to the consideration of travel time correlations and reliability. Consequently, traditional methods for identifying critical links cannot be directly applied in this context. In light of this, a modified heuristic algorithm is presented based on the algorithm proposed by Shen et al. [20, 26]. On the basis of the above discussion and the algorithm proposed in [20, 26], we try to transform the objective function of the proposed optimization model into two parts, in which both of them satisfies the property of additive. Under this circumstance, Theorem 1 is proposed to find the upper and lower bounds of the objective function (7), in which the deduced upper and lower bounds satisfies the additive property.

**Theorem 1.** Inspired by Shen et al. [20], the upper and lower bounds of the objective function can be obtained as follows (*θ* ≥ 50%):

(1) Upper bound:

$$\underset{rs}{\sum }\underset{a\in E}{\sum }\min t_{k}^{rs}(\Delta \mu_{a},\Delta \sigma_{a})<\sum_{rs}\left(\underset{a\in E}{\sum }\delta_{a,k}^{rs}\Delta \mu_{a}+\Phi^{-1}(\theta )\overset{23}{\underset{a,b\in E}{\sum }}\delta_{a,k}^{rs}\delta_{b,k}^{rs}(\Delta \sigma_{a}+\Delta \sigma_{b})\right)=UB$$ (8)

(2) Lower bound:

$$\underset{rs}{\sum }\underset{a\in E}{\sum }\min t_{k}^{rs}(\Delta \mu_{a},\Delta \sigma_{a})>\sum_{rs}\underset{a\in E}{\sum }\delta_{a,k}^{rs}\Delta \mu_{a}=LB$$ (9)

**Proof.** The detailed process can be found in Shen et al. [20]. This is the completion of the proof.

Based on the Theorem 1, the upper and lower bounds of the objective function can be obtained. Moreover, the *UB* and *LB* satisfies the property of additive. Therefore, we can use the traditional Dijkstra algorithm to obtain the minimum value of *UB* firstly and regarded it as $k_{\min}^{UB}$, and the corresponding effective travel time is expressed as ${\tilde{t}}_{k,temp}$. Secondly, we can use K-shortest algorithm [20] to obtain the first K minimum values of *LB* and denoted it as $k_{x}^{LB}(x=1,2,\cdots ,K)$. On the basis of Shen et al. [20], if the $k_{x}^{LB}<{\tilde{t}}_{k,temp}(x=1,2\cdots ,K)$, then the path *k*_*x*_ can be put into the candidate path set **Q**. The detailed solution algorithm is depicted as follows:

### 3.1. Algorithm steps

**Step 1:** Initialization, we denote *Tem* = inf, *i* = 1;
**Step 2:** Initialization, *Vs* = 1, *Vd* = 1, *Total* = 0;
**Step 3:** To determine if two values *Vs* and *Vd* are equal? If they are not equal, proceed to step 4. Otherwise, proceed to step 5;
**Step 4:** Calculating the reliable paths and effective travel time Re_*t* for each OD pair (*Vs*,*Vd*) using the algorithm proposed by Shen et al. [20, 26]; Sum up the reliable travel time for each OD pair to obtain the total travel time *Total* = *Total* + Re_*t* for the entire network;
**Step 5:** For *Vd* = *Vd*+1, if *Vd* > *NofL*, go to step 6. Otherwise, go to step 3;
**Step 6:** For *Vs* = *Vs*+1, if *Vs* > *NofL*, go to step 7. Otherwise, go to step 3;
**Step 7:** If *Total* < *Tem*, execute *Tem* = *Total*. Otherwise, go to step 8;
**Step 8:** If *Vd* > 76 and *Vs* > 76 are true, output the total travel time *Tem* and link *i*. Otherwise, to reset the mean and variance matrices of the network to their initial state, we can reduce the mean and variance of link *i* by half, then *i* = *i*+1 and go to step 2.

In this algorithm, we make full use of the reliable path search algorithm based on scaling proposed by Shen et al. [20, 26], which significantly improves the efficiency of the algorithm. By providing the total travel time for the entire network, it demonstrates the accuracy of the results.

### 3.2. The flowchart of the heuristic algorithm

**Fig 2:**
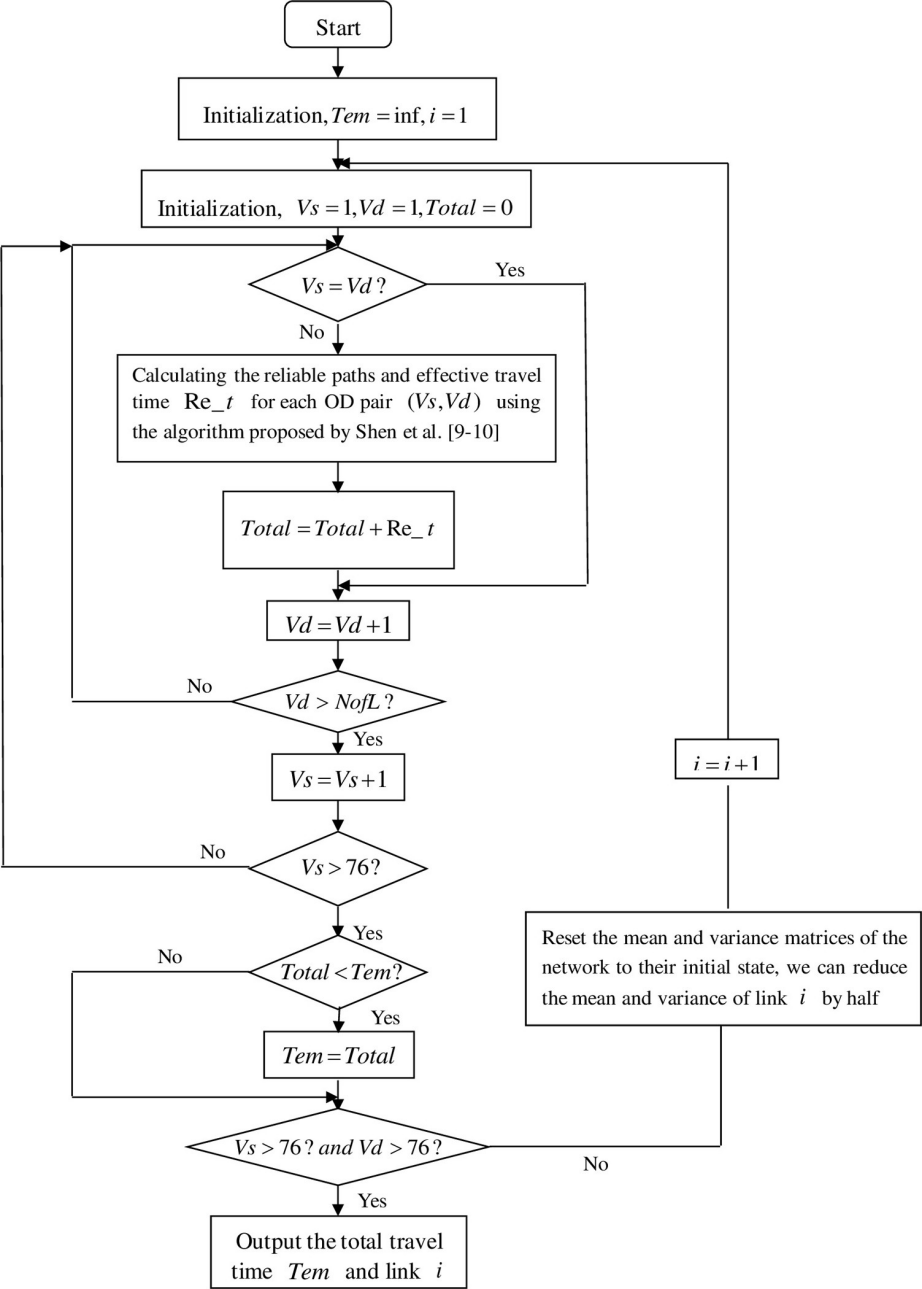
The flowchart of the heuristic algorithm.

To visually depict the detailed process of the algorithm, a flowchart has been created based on the steps outlined in section 3.1 of the proposed algorithm. The flowchart (Fig 2) provides a clear and concise representation of the algorithm’s progression.

## 4. Numerical examples

To illustrate the improved search algorithm for finding the optimal path with consideration of link correlations under uncertainty, we will work through two examples: a small network with 9 nodes and a medium-sized network with 76 nodes. We will follow the step-by-step process outlined earlier to execute the algorithm.

### 4.1. A small traffic network

**Fig 3:**
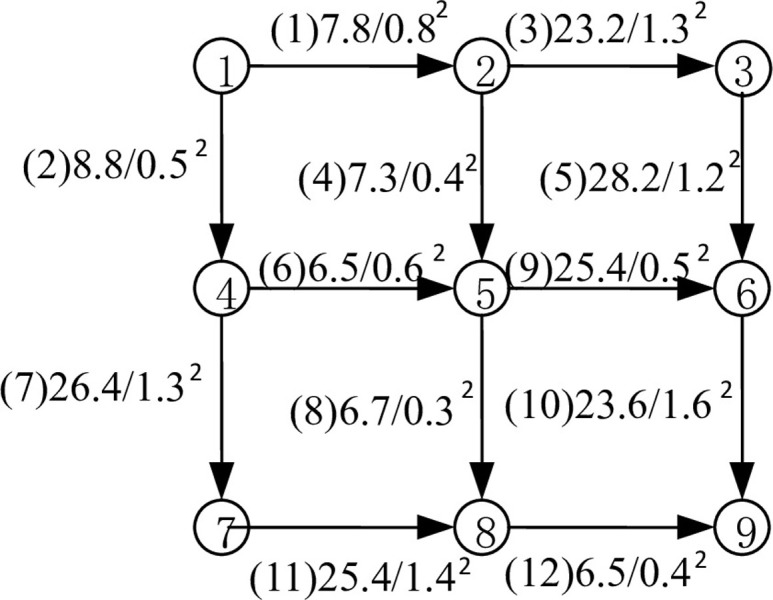
The simple network under uncertainty.

In this study, we analyze a small-scale transportation network depicted in Fig 3, where uncertain conditions can affect the link travel time. We assume that the travel times follow a normal distribution with mean and variance values indicated in the diagram. Node 1 serves as the original node, while node 9 acts as the destination node. Given a specified reliability level, the link correlations are shown in Table 1, which contains a symmetric matrix of correlation coefficients for each link.

**Table 1:** The correlation coefficient matrix.

Link	(1)	(2)	(3)	(4)	(5)	(6)	(7)	(8)	(9)	(10)	(11)	(12)
(1)	1	0.02	0.46	0.21	0.35	0.05	-0.81	-0.64	-0.64	-0.18	0.55	-0.26
(2)	0.02	1	-0.52	-0.13	0.02	0.58	-0.06	-0.43	0.12	0.05	0.21	0.13
(3)	0.46	-0.52	1	-0.06	0.67	-0.11	-0.20	-0.60	-0.30	-0.55	-0.24	-0.40
(4)	0.21	-0.13	-0.06	1	-0.50	-0.07	0.06	0.09	0.07	-0.53	-0.02	0.03
(5)	0.35	0.02	0.67	-0.50	1	0.07	-0.24	0.06	-0.02	0.21	0.09	-0.01
(6)	0.05	0.58	-0.11	-0.07	0.07	1	0.16	-0.05	0.40	0.11	-0.46	0.11
(7)	-0.81	-0.06	-0.20	0.06	-0.24	0.16	1	0.31	0.21	-0.04	0.19	0.17
(8)	-0.64	-0.43	-0.60	0.09	0.06	-0.05	0.31	1	0.17	0.62	0.79	-0.48
(9)	-0.64	0.12	-0.30	0.07	-0.02	0.40	0.21	0.17	1	0.10	-0.05	0.02
(10)	-0.18	0.05	-0.55	-0.53	0.21	0.11	-0.04	0.62	0.10	1	0.28	0.12
(11)	0.55	0.21	-0.24	-0.02	0.09	-0.46	0.19	0.79	-0.05	0.28	1	0.60
(12)	-0.26	0.13	-0.40	0.03	-0.01	0.11	0.17	-0.48	0.02	0.12	0.60	1

The detailed solution process is as follows:

First, we provide the effective travel time for each pair of optimal reliable paths in a small-scale transportation network under uncertain conditions. The results are shown in Table 2:

**Table 2:** The effective travel time of optimal path of different *OD* pairs.

*OD* pair	Effective travel time (minutes)	Path	*OD* pair	Effective travel time (minutes)	Path
[1,2]	8.4733	[1,2]	[3,9]	53.6478	[3,6,9]
[1,3]	32.2968	[1,2,3]	[4,5]	7.0050	[4,5]
[1,4]	9.2208	[1,4]	[4,6]	32.6066	[4,5,6]
[1,5]	15.9126	[1,2,5]	[4,7]	27.4941	[4,7]
[1,6]	41.1265	[1,2,5,6]	[4,8]	13.8490	[4,5,8]
[1,7]	36.1088	[1,4,7]	[4,9]	20.4581	[4,5,8,9]
[1,8]	22.5209	[1,2,5,8]	[5,6]	25.8208	[5,6]
[1,9]	29.0243	[1,2,5,8,9]	[5,8]	6.9525	[5,8]
[2,3]	24.2941	[2,3]	[5,9]	13.6246	[5,8,9]
[2,5]	7.6366	[2,5]	[6,9]	24.9466	[6,9]
[2,6]	33.2533	[2,5,6]	[7,8]	26.5783	[7,8]
[2,8]	14.4351	[2,5,8]	[7,9]	33.3070	[7,8,9]
[2,9]	21.0582	[2,5,8,9]	[8,9]	6.8366	[8,9]
[3,6]	29.2099	[3,6]			

According to the algorithm steps in this section, we need to calculate the total system time for the entire network. Then, based on the improvement of different road sections, we can determine the optimal results. The results are shown in Table 3:

**Table 3:** The improved system total travel time.

Number of improved link	System total travel time (minutes)	Optimized results
Results without making any changes to links	**617.6984**	/
Link 1	593.4632	24.2352
Link 2	591.7642	25.9342
Link 3	593.9175	23.7809
Link 4	587.9500	29.7484
Link 5	589.0083	28.6901
Link 6	591.4667	26.2317
Link 7	590.6920	27.0064
Link 8	590.6527	27.0457
Link 9	**566.6345**	51.0639
Link 10	593.3851	24.3133
Link 11	591.6165	26.0819
Link 12	597.8339	19.8645

The optimal results obtained in Table 3 consist of two main parts. For comparison, the first part is presented to show the system total travel time without making any changes to links (row 2). The second part is the system total travel times under the condition of reducing the mean and variance travel time of link *i*(*i* = 1,2,⋯,9) by half (rows 3 to 14). From Table 3, it can be observed that the road section No. 9 is the most improved link with the minimum total system time (566.6345 minutes) if reduce the mean and variance travel time of link *i*(*i* = 1,2,⋯,9) by half. That is to say, it is the most effective improvement among all the 12 road sections. In addition, if policy makers want to improve the traffic capacity of the entire network, then the road expansion of section 9 is a better choice. That is, spend the same amount of money, get the maximum benefit. Under this circumstance, the road section No. 9 is a critical link for this simple network.

### 4.2. A medium-sized transportation network

**Fig 4:**
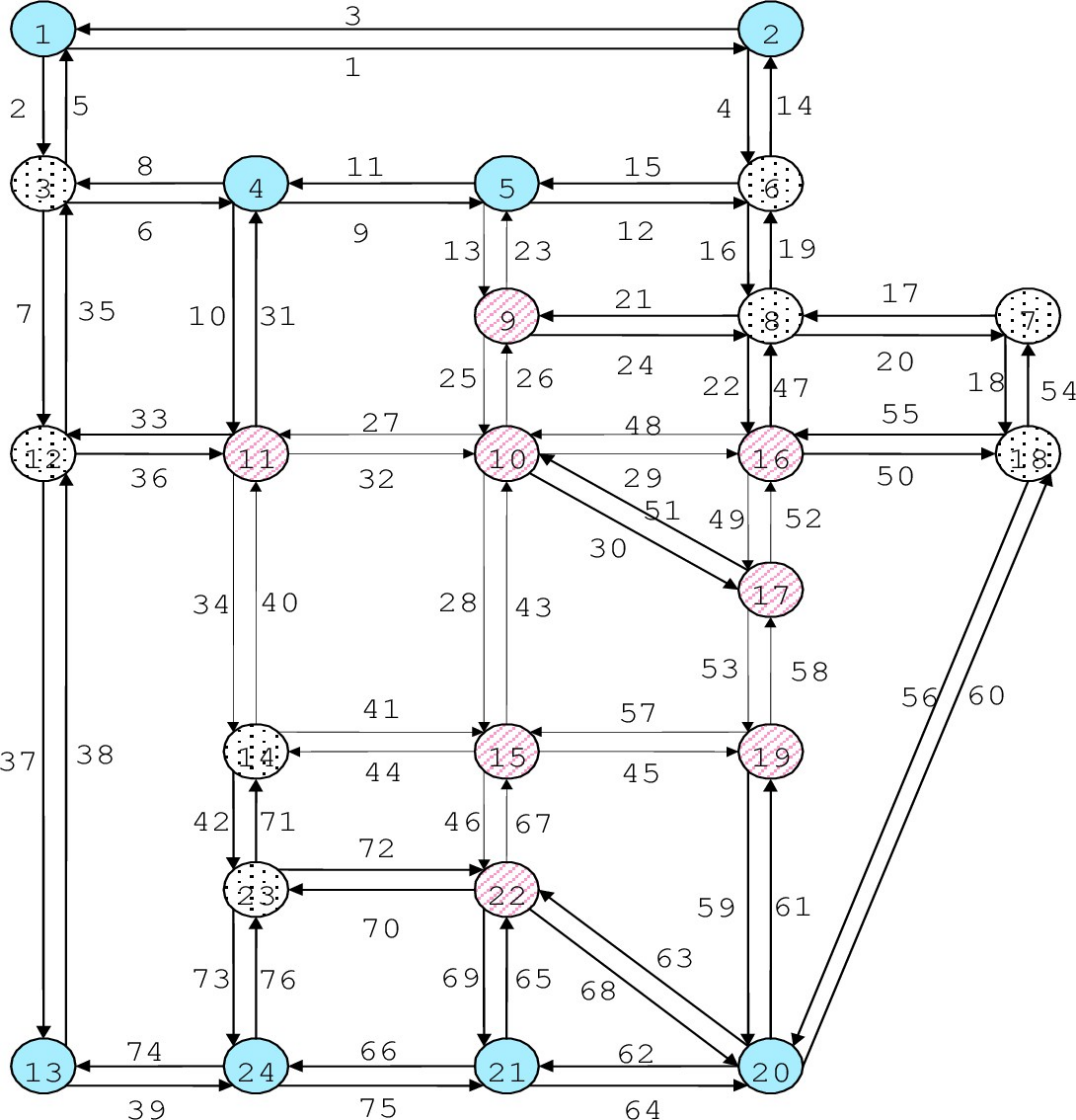
Sioux falls transportation network.

Fig 4 is a medium-scale transportation network graph under uncertainty conditions. Assuming that the travel times of each link follow a normal distribution and considering the level of reliability, the improved search algorithm is adopted to find the optimal result.

The detailed solution process is as follows. Firstly, we calculate the total system time of the medium-scale network graph in its initial state.


$$Tem\_Totaltime=5.829\times 10^{4}$$

**Fig 5:**
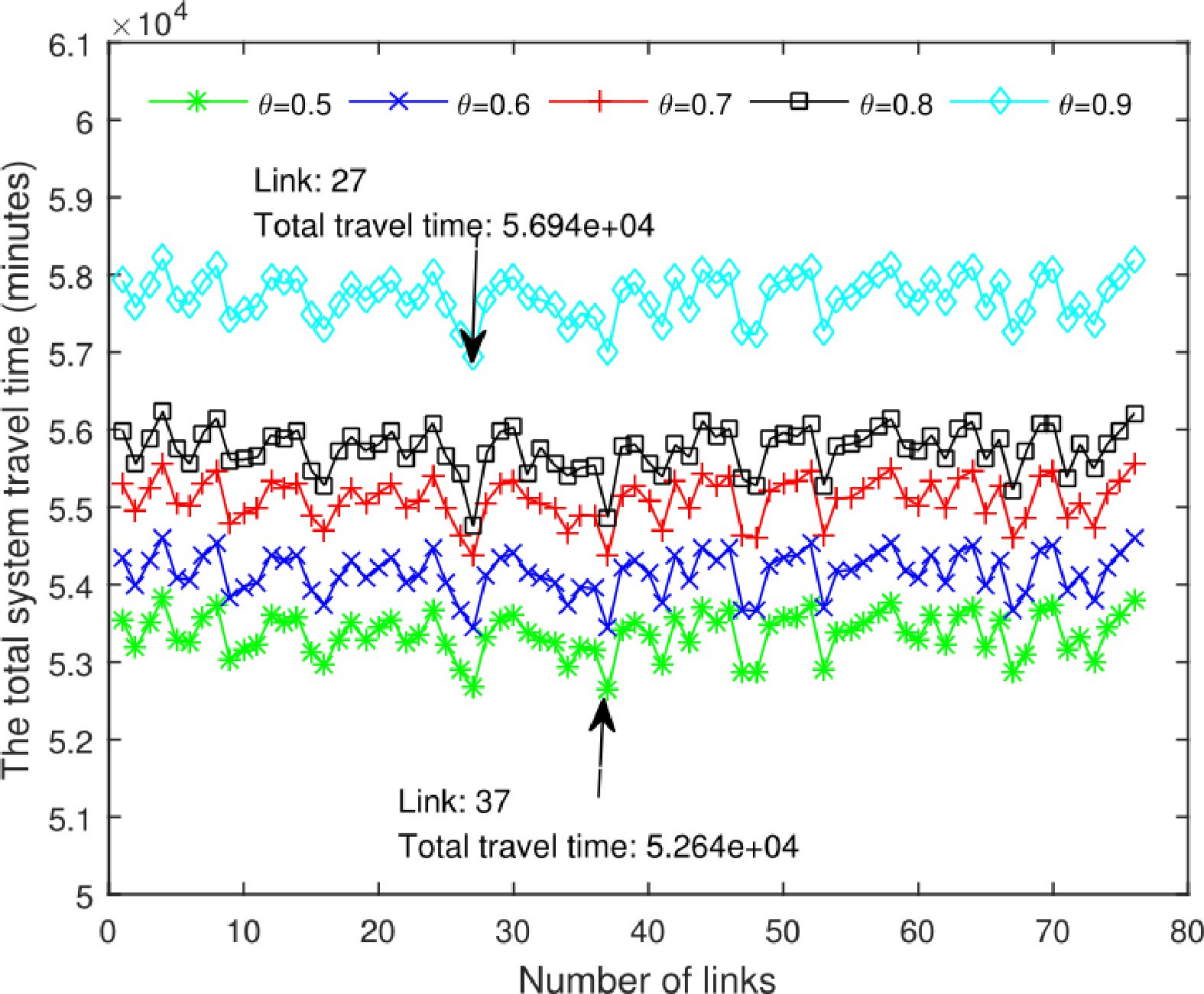
Results of the proposed method.

Afterwards, we proceed to improve each road section in turn, and obtain the total system time after improving each road section under different values of reliability. The specific results are shown in Fig 5.

According to the results shown in Fig 5, it can be observed that the total system travel time obtained after improving different road sections is not greater than the total system time before improvement. Specifically, for road section 4, the total system time remains the same after improvement, indicating that the improvement on road section 4 did not alleviate the traffic congestion. This explains why in real life, improving a certain road section may not necessarily improve the overall traffic conditions. Furthermore, it is evident that the total system time is minimized after improving road section 27 with on-time reliability *θ* = 0.9. This indicates that the improvement on road section 27 has the most significant effect in alleviating traffic congestion. However, the total system time is minimized after improving road section 37 with on-time reliability *θ* = 0.5 (without consideration of travel time variance). This shows that if the travel time reliability is not considered, it will bring errors to the identification of key links.

Based on the two different-scale network graphs mentioned above, the overall running time of the algorithm is provided as follows:

From Table 4, the computational time for the small transportation network is 0.664 seconds and for the for the small transportation network is 250.293 seconds. This results indicate that as the number of nodes increases, the running time of the algorithm also tends to increase correspondingly. This is because more nodes imply more paths and possible combinations, leading to the algorithm requiring more computations and comparisons to find the optimal solution. Larger-scale network graphs typically require more computational resources and time to complete the execution of the algorithm. However, the computational time of the proposed algorithm is within a reasonable range, hence we think it is an efficient algorithm.

**Table 4:** The algorithm running time.

Scale of networks	Average running time (seconds)
A small transportation network (9 nodes and 12 links)	0.664000
A medium-sized transportation network (24 nodes and 76 links)	250.293000

## 5. Conclusions

We propose a new optimization mathematical model to identify crucial links in urban road transportation networks. This model focuses on selecting the best path for each origin-destination pair under uncertain conditions, taking into account the link correlations. By estimating the time needed for all pairs in the network, the model aims enhance the overall network performance by minimizing the time required for all pairs through improvements made to links. Considering the stochastic nature of origin-destination demand, link travel time is treated as a random variable. To account for both mean travel time and reliability, we introduce the concept of effective travel time, which includes a safety margin. Our numerical examples in Section 4 illustrate the effectiveness of the model for medium-sized transportation networks, offering valuable insights for optimizing urban road transportation systems. The calculation results show that: (1) The travel time reliability is an important factor for the identification of critical links in stochastic traffic networks. If it is not considered, it will bring errors to the identification of key links. (2) The solution algorithm proposed in this paper offers a method to bypass the necessity of conducting a full scan of the entire road network. (3) The computational time of the proposed algorithm is within a reasonable range and hence we think it is an efficient algorithm.

Overall, our proposed optimization model based on optimal reliable paths offers a promising approach to identify critical links in urban road transportation, accounting for uncertainty and improving network performance.

However, this paper has several limitations, and thus, we suggest the following future research works.

(1) The proposed reliability-based path-finding model is a static model that calculates effective travel times in stochastic road networks. Extending this model to a dynamic setting (Unal et al. [27]), considering time-varying stochastic networks, shows great promise for further research.
(2) To validate the applicability of our proposed algorithm, it is worthwhile to construct a large-scale transportation network in future research (Yu et al. [28]).
(3) To verify the effectiveness of the proposed algorithm, some important indicators, such as network capacity, network efficiency, and link capacity are deserved to adopted in the future research.

## Supporting information

S1 Data The mean and variance travel time for each link. (DOCX)
