# Supplementary material for: Identification of critical links based on the optimal reliable path in stochastic traffic networks
Source: PLoS One. 2024 Apr 9;19(4):e0301272. doi: 10.1371/journal.pone.0301272 (PMC11003686; doi:10.1371/journal.pone.0301272)
Supplement: S1 Data — (DOCX) [file pone.0301272.s001.docx]

| Inf | 55.1 | 34.5 | Inf | Inf | Inf | Inf | Inf | Inf | Inf | Inf | Inf | Inf | Inf | Inf | Inf | Inf | Inf | Inf | Inf | Inf | Inf | Inf | Inf |
| --- | --- | --- | --- | --- | --- | --- | --- | --- | --- | --- | --- | --- | --- | --- | --- | --- | --- | --- | --- | --- | --- | --- | --- |
| 26.8 | Inf | Inf | Inf | Inf | 28.4 | Inf | Inf | Inf | Inf | Inf | Inf | Inf | Inf | Inf | Inf | Inf | Inf | Inf | Inf | Inf | Inf | Inf | Inf |
| 49.0 | Inf | Inf | 22.0 | Inf | Inf | Inf | Inf | Inf | Inf | Inf | 14.8 | Inf | Inf | Inf | Inf | Inf | Inf | Inf | Inf | Inf | Inf | Inf | Inf |
| Inf | Inf | 57.1 | Inf | 38.7 | Inf | Inf | Inf | Inf | Inf | 21.7 | Inf | Inf | Inf | Inf | Inf | Inf | Inf | Inf | Inf | Inf | Inf | Inf | Inf |
| Inf | Inf | Inf | 51.0 | Inf | 12.1 | Inf | Inf | 42.4 | Inf | Inf | Inf | Inf | Inf | Inf | Inf | Inf | Inf | Inf | Inf | Inf | Inf | Inf | Inf |
| Inf | 42.3 | Inf | Inf | 37.3 | Inf | Inf | 47.2 | Inf | Inf | Inf | Inf | Inf | Inf | Inf | Inf | Inf | Inf | Inf | Inf | Inf | Inf | Inf | Inf |
| Inf | Inf | Inf | Inf | Inf | Inf | Inf | 44.3 | Inf | Inf | Inf | Inf | Inf | Inf | Inf | Inf | Inf | 28.4 | Inf | Inf | Inf | Inf | Inf | Inf |
| Inf | Inf | Inf | Inf | Inf | 49.0 | 56.4 | Inf | 34.3 | Inf | Inf | Inf | Inf | Inf | Inf | 32.3 | Inf | Inf | Inf | Inf | Inf | Inf | Inf | Inf |
| Inf | Inf | Inf | Inf | 35.4 | Inf | Inf | 50.8 | Inf | 42.2 | Inf | Inf | Inf | Inf | Inf | Inf | Inf | Inf | Inf | Inf | Inf | Inf | Inf | Inf |
| Inf | Inf | Inf | Inf | Inf | Inf | Inf | Inf | 50.5 | Inf | 27.5 | Inf | Inf | Inf | 53.7 | 41.1 | 20.3 | Inf | Inf | Inf | Inf | Inf | Inf | Inf |
| Inf | Inf | Inf | 33.5 | Inf | Inf | Inf | Inf | Inf | 52.2 | Inf | 21.2 | Inf | 21.3 | Inf | Inf | Inf | Inf | Inf | Inf | Inf | Inf | Inf | Inf |
| Inf | Inf | 25.5 | Inf | Inf | Inf | Inf | Inf | Inf | Inf | 31.5 | Inf | 55.2 | Inf | Inf | Inf | Inf | Inf | Inf | Inf | Inf | Inf | Inf | Inf |
| Inf | Inf | Inf | Inf | Inf | Inf | Inf | Inf | Inf | Inf | Inf | 31.9 | Inf | Inf | Inf | Inf | Inf | Inf | Inf | Inf | Inf | Inf | Inf | 22.9 |
| Inf | Inf | Inf | Inf | Inf | Inf | Inf | Inf | Inf | Inf | 39.7 | Inf | Inf | Inf | 40.1 | Inf | Inf | Inf | Inf | Inf | Inf | Inf | 21.0 | Inf |
| Inf | Inf | Inf | Inf | Inf | Inf | Inf | Inf | Inf | 24.8 | Inf | Inf | Inf | 31.2 | Inf | Inf | Inf | Inf | 14.2 | Inf | Inf | 50.0 | Inf | Inf |
| Inf | Inf | Inf | Inf | Inf | Inf | Inf | 56.4 | Inf | 34.4 | Inf | Inf | Inf | Inf | Inf | Inf | 21.8 | 58.1 | Inf | Inf | Inf | Inf | Inf | Inf |
| Inf | Inf | Inf | Inf | Inf | Inf | Inf | Inf | Inf | 36.0 | Inf | Inf | Inf | Inf | Inf | 34.4 | Inf | Inf | 43.9 | Inf | Inf | Inf | Inf | Inf |
| Inf | Inf | Inf | Inf | Inf | Inf | 28.3 | Inf | Inf | Inf | Inf | Inf | Inf | Inf | Inf | 11.8 | Inf | Inf | Inf | 55.6 | Inf | Inf | Inf | Inf |
| Inf | Inf | Inf | Inf | Inf | Inf | Inf | Inf | Inf | Inf | Inf | Inf | Inf | Inf | 14.9 | Inf | 26.7 | Inf | Inf | 16.8 | Inf | Inf | Inf | Inf |
| Inf | Inf | Inf | Inf | Inf | Inf | Inf | Inf | Inf | Inf | Inf | Inf | Inf | Inf | Inf | Inf | Inf | 15.3 | 34.7 | Inf | 45.7 | 54.5 | Inf | Inf |
| Inf | Inf | Inf | Inf | Inf | Inf | Inf | Inf | Inf | Inf | Inf | Inf | Inf | Inf | Inf | Inf | Inf | Inf | Inf | 44.9 | Inf | 11.5 | Inf | 35.0 |
| Inf | Inf | Inf | Inf | Inf | Inf | Inf | Inf | Inf | Inf | Inf | Inf | Inf | Inf | 55.2 | Inf | Inf | Inf | Inf | 40.8 | 50.2 | Inf | 19.1 | Inf |
| Inf | Inf | Inf | Inf | Inf | Inf | Inf | Inf | Inf | Inf | Inf | Inf | Inf | 54.3 | Inf | Inf | Inf | Inf | Inf | Inf | Inf | 34.4 | Inf | 58.9 |
| Inf | Inf | Inf | Inf | Inf | Inf | Inf | Inf | Inf | Inf | Inf | Inf | 35.0 | Inf | Inf | Inf | Inf | Inf | Inf | Inf | 12.9 | Inf | 12.1 | Inf |

Table A.1. The mean travel time for each link

| Inf | 22.3 | 5.98 | Inf | Inf | Inf | Inf | Inf | Inf | Inf | Inf | Inf | Inf | Inf | Inf | Inf | Inf | Inf | Inf | Inf | Inf | Inf | Inf | Inf |
| --- | --- | --- | --- | --- | --- | --- | --- | --- | --- | --- | --- | --- | --- | --- | --- | --- | --- | --- | --- | --- | --- | --- | --- |
| 20.2 | Inf | Inf | Inf | Inf | 0.30 | Inf | Inf | Inf | Inf | Inf | Inf | Inf | Inf | Inf | Inf | Inf | Inf | Inf | Inf | Inf | Inf | Inf | Inf |
| 3.79 | Inf | Inf | 4.07 | Inf | Inf | Inf | Inf | Inf | Inf | Inf | 0.43 | Inf | Inf | Inf | Inf | Inf | Inf | Inf | Inf | Inf | Inf | Inf | Inf |
| Inf | Inf | 22.8 | Inf | 0.08 | Inf | Inf | Inf | Inf | Inf | 3.11 | Inf | Inf | Inf | Inf | Inf | Inf | Inf | Inf | Inf | Inf | Inf | Inf | Inf |
| Inf | Inf | Inf | 0.01 | Inf | 0.71 | Inf | Inf | 13.3 | Inf | Inf | Inf | Inf | Inf | Inf | Inf | Inf | Inf | Inf | Inf | Inf | Inf | Inf | Inf |
| Inf | 5.08 | Inf | Inf | 2.19 | Inf | Inf | 0.89 | Inf | Inf | Inf | Inf | Inf | Inf | Inf | Inf | Inf | Inf | Inf | Inf | Inf | Inf | Inf | Inf |
| Inf | Inf | Inf | Inf | Inf | Inf | Inf | 0.84 | Inf | Inf | Inf | Inf | Inf | Inf | Inf | Inf | Inf | 9.78 | Inf | Inf | Inf | Inf | Inf | Inf |
| Inf | Inf | Inf | Inf | Inf | 0.16 | 15.0 | Inf | 4.74 | Inf | Inf | Inf | Inf | Inf | Inf | 2.34 | Inf | Inf | Inf | Inf | Inf | Inf | Inf | Inf |
| Inf | Inf | Inf | Inf | 6.52 | Inf | Inf | 15.7 | Inf | 3.58 | Inf | Inf | Inf | Inf | Inf | Inf | Inf | Inf | Inf | Inf | Inf | Inf | Inf | Inf |
| Inf | Inf | Inf | Inf | Inf | Inf | Inf | Inf | 7.09 | Inf | 22.0 | Inf | Inf | Inf | 7.56 | 8.61 | 2.26 | Inf | Inf | Inf | Inf | Inf | Inf | Inf |
| Inf | Inf | Inf | 1.32 | Inf | Inf | Inf | Inf | Inf | 0.94 | Inf | 0.72 | Inf | 4.74 | Inf | Inf | Inf | Inf | Inf | Inf | Inf | Inf | Inf | Inf |
| Inf | Inf | 21.3 | Inf | Inf | Inf | Inf | Inf | Inf | Inf | 0.85 | Inf | 23.9 | Inf | Inf | Inf | Inf | Inf | Inf | Inf | Inf | Inf | Inf | Inf |
| Inf | Inf | Inf | Inf | Inf | Inf | Inf | Inf | Inf | Inf | Inf | 0.30 | Inf | Inf | Inf | Inf | Inf | Inf | Inf | Inf | Inf | Inf | Inf | 4.17 |
| Inf | Inf | Inf | Inf | Inf | Inf | Inf | Inf | Inf | Inf | 1.71 | Inf | Inf | Inf | 12.6 | Inf | Inf | Inf | Inf | Inf | Inf | Inf | 0.34 | Inf |
| Inf | Inf | Inf | Inf | Inf | Inf | Inf | Inf | Inf | 2.54 | Inf | Inf | Inf | 6.44 | Inf | Inf | Inf | Inf | 1.72 | Inf | Inf | 0.02 | Inf | Inf |
| Inf | Inf | Inf | Inf | Inf | Inf | Inf | 13.3 | Inf | 8.36 | Inf | Inf | Inf | Inf | Inf | Inf | 5.26 | 7.47 | Inf | Inf | Inf | Inf | Inf | Inf |
| Inf | Inf | Inf | Inf | Inf | Inf | Inf | Inf | Inf | 1.34 | Inf | Inf | Inf | Inf | Inf | 9.73 | Inf | Inf | 3.91 | Inf | Inf | Inf | Inf | Inf |
| Inf | Inf | Inf | Inf | Inf | Inf | 24.4 | Inf | Inf | Inf | Inf | Inf | Inf | Inf | Inf | 19.5 | Inf | Inf | Inf | 15.8 | Inf | Inf | Inf | Inf |
| Inf | Inf | Inf | Inf | Inf | Inf | Inf | Inf | Inf | Inf | Inf | Inf | Inf | Inf | 1.71 | Inf | 11.5 | Inf | Inf | 13.0 | Inf | Inf | Inf | Inf |
| Inf | Inf | Inf | Inf | Inf | Inf | Inf | Inf | Inf | Inf | Inf | Inf | Inf | Inf | Inf | Inf | Inf | 10.6 | 15.1 | Inf | 20.4 | 2.79 | Inf | Inf |
| Inf | Inf | Inf | Inf | Inf | Inf | Inf | Inf | Inf | Inf | Inf | Inf | Inf | Inf | Inf | Inf | Inf | Inf | Inf | 0.97 | Inf | 13.8 | Inf | 5.75 |
| Inf | Inf | Inf | Inf | Inf | Inf | Inf | Inf | Inf | Inf | Inf | Inf | Inf | Inf | 9.29 | Inf | Inf | Inf | Inf | 18.4 | 8.31 | Inf | 1.43 | Inf |
| Inf | Inf | Inf | Inf | Inf | Inf | Inf | Inf | Inf | Inf | Inf | Inf | Inf | 0.02 | Inf | Inf | Inf | Inf | Inf | Inf | Inf | 0.70 | Inf | 12.6 |
| Inf | Inf | Inf | Inf | Inf | Inf | Inf | Inf | Inf | Inf | Inf | Inf | 5.54 | Inf | Inf | Inf | Inf | Inf | Inf | Inf | 11.6 | Inf | 0.12 | Inf |

Table A.2. The variance travel time for each link
